# Effectiveness of Combined Cognitive Stimulation and Physical Activity Interventions on Activities of Daily Living, Cognitive Function, and Physical Function in Older People with Mild Cognitive Impairment: A Systematic Review with Meta-Analysis

**DOI:** 10.3390/jcm14072261

**Published:** 2025-03-26

**Authors:** Edgar Vásquez-Carrasco, Celia Sánchez Gómez, Pablo Valdés-Badilla, Jordan Hernandez-Martinez, Francisca Villagrán-Silva, Pablo Aravena-Sagardia, Cristian Sandoval, Pedro Moruno Miralles

**Affiliations:** 1 School of Occupational Therapy, Faculty of Psychology, Universidad de Talca, Talca 3465548, Chile; edgar.vasquez@utalca.cl; 2 Centro de Investigación en Ciencias Cognitivas, Faculty of Psychology, Universidad de Talca, Talca 3465548, Chile; 3 Department of Developmental and Educational Psychology, Universidad de Salamanca, 37008 Salamanca, Spain; 4 Institute of Biomedical Research of Salamanca (IBSAL), 37008 Salamanca, Spain; 5 Department of Physical Activity Sciences, Faculty of Education Sciences, Universidad Católica del Maule, Talca 3530000, Chile; 6 Sports Coach Career, School of Education, Universidad Viña del Mar, Viña del Mar 2520000, Chile; 7 Department of Physical Activity Sciences, Universidad de Los Lagos, Osorno 5290000, Chile; jordan.hernandez@ulagos.cl; 8 G-IDyAF Research Group, Department of Physical Activity Sciences, Universidad de Los Lagos, Osorno 5290000, Chile; 9 Programa de Investigación en Deporte, Sociedad y Buen Vivir, Universidad de los Lagos, Osorno 5290000, Chile; 10 Programa de Doctorado en Ciencias Morfológicas, Facultad de Medicina, Universidad de La Frontera, Temuco 4811230, Chile; f.villagran04@ufromail.cl; 11 Physical Education Pedagogy, Faculty of Education, Universidad Autónoma de Chile, Temuco 4811230, Chile; pablo.aravena@uautonoma.cl; 12 Escuela de Tecnología Médica, Facultad de Salud, Universidad Santo Tomás, Los Carreras 753, Osorno 5310431, Chile; 13 Departamento de Medicina Interna, Facultad de Medicina, Universidad de La Frontera, Temuco 4811230, Chile; 14 Núcleo Científico y Tecnológico en Biorecursos (BIOREN), Universidad de La Frontera, Temuco 4811230, Chile; 15 Department of Nursing, Physiotherapy and Occupational Therapy, University of Castilla-La Mancha, 45600 Toledo, Spain; pedro.moruno@uclm.es

**Keywords:** cognitive dysfunction, physical fitness, cognition, aging, trail making test

## Abstract

**Background/Objectives:** This systematic review with meta-analysis aimed to evaluate and synthesize scientific evidence about the combined cognitive stimulation and physical activity interventions on Activities of Daily Living (ADL), cognitive function, and physical function in older people with Mild Cognitive Impairment (MCI). **Methods:** A systematic literature search was conducted between August 2024 and October 2024 using the core collection of six generic databases: PubMed, EBSCOhost, CINAHL Complete, Cochrane, Scopus, and Web of Science. The PRISMA, RoB 2, and GRADEpro tools assessed the evidence’s methodological quality and certainty. The protocol was registered in PROSPERO, CRD42024577229. **Results:** Of 270 records identified in the databases, 9 studies were analyzed using the PICOS format. The MMSE meta-analysis showed significant improvements in cognitive function in favor of the experimental groups (*p* = 0.010). In contrast, no significant improvements were found for TMT-A (*p* = 0.51) and TMT-B (*p* = 0.37). No significant differences were reported for the other variables studied. **Conclusions**: Cognitive function, as measured by the MMSE, showed significant improvements, while the interventions analyzed did not produce significant improvements in ADL or physical function among older people with MCI. Individual studies suggest that programs that integrate both cognitive stimulation and physical activity components may provide some benefits.

## 1. Introduction

In older people, regular physical activity improves mental health and cognitive function and reduces the risks of MCI, dementia, depression, and anxiety [1]. It enhances muscle strength, balance, and cardiorespiratory fitness, thus aiding in independence, fall prevention, and reducing chronic disease risks [2]. Physically active lifestyles significantly improve cognitive functions (*p* < 0.01), notably memory and executive abilities, by promoting neurogenesis, increasing hippocampal volume, and enhancing neural connectivity, facilitated by increased brain-derived neurotrophic factor levels [3,4,5]. A meta-analysis supports that interventions by way of cognitive stimulation and physical activity significantly benefit cognitive function in older people, with or without MCI [6]. Another meta-analysis corroborates that the combination of physical exercise and cognitive training improves memory (*p* < 0.0001), executive functions (*p* < 0.00001), and physical skills (*p* = 0.03); these interventions also favor neurogenesis and improve cognitive function [7].

On the other hand, cognitive stimulation through activities such as memory games and mental exercises can slow down cognitive decline and improve quality of life in people with dementia or MCI [8]. A meta-analysis supports that combining physical and cognitive activities effectively enhances overall well-being and mental health in older people (*p* < 0.001) [9]. Occupational therapy (OT) further supports these goals, aiding skill recovery, mental health, and adaptation to new daily functioning [10]. OT employs creative activities to improve mood and uses environmental modifications and assistive tech to enable daily life engagement [11,12]. Group exercise in OT not only enhances physical fitness but also combats social isolation, thus promoting community and reducing depressive symptoms [13]. Studies show that OT interventions, combining physical and cognitive activities, enhance quality of life, cognitive health, and functional independence [14,15].

Specific studies emphasize the combined benefit of physical activity and diet on cognitive health [16], with exercise–cognitive programs reducing depression and anxiety [17]. Targeted programs for MCI populations enhance memory and executive function [18], and individualized interventions improve quality of life and independence [19]. Although there is strong evidence that occupational therapy improves functioning in frail older adults, there is still a lack of clarity regarding the optimal dosage and specific nature of the interventions [20]. Therefore, this systematic review with meta-analysis aimed to evaluate and synthesize scientific evidence about the combined cognitive stimulation and physical activity interventions on ADL, cognitive function, and physical function in older people with MCI.

## 2. Materials and Methods

### 2.1. Protocol and Registration

This systematic review with meta-analysis follows the Cochrane Collaboration methodology [21] and adheres to the PRISMA checklist and flowchart standards for reporting [22]. The review protocol is registered in the PROSPERO database under identification code CRD42024577229.

### 2.2. Eligibility Criteria

This systematic review with meta-analysis includes peer-reviewed original articles, specifically randomized controlled trials (RCTs), with no restrictions on language or publication date, up to October 2024. Excluded materials include conference abstracts, books and book chapters, editorials, letters to the editor, protocol records, reviews, case studies, and non-randomized trials. The PICOS (Population, Intervention, Comparator, Outcome, Study design) framework is used to guide the inclusion of studies (Table 1).

### 2.3. Information Search Process and Databases

Five databases were consulted: Scopus, Web of Science (core collection), Medline/PubMed, EBSCOhost, and the Cochrane Library. The US National Library of Medicine’s Medical Subject Headings (MeSH) and free language phrases related to OT, physical activity, cognitive stimulation, and older people were used. The following was used: (“physical exercise” OR “endurance” OR “physical fitness” OR “physical performance” OR “physical function” OR “physical activity” OR “exercise” OR “resistance training” OR “physical exercise” OR “walking” OR “strength training” OR “running” OR “sports” OR “sport”) AND (“cognitive training” OR “electroencephalography” OR “psychomotor performance” OR “neuropsychological tests” OR “cognition” OR “executive function” OR “brain function” OR “cognitive process” OR “cognitive processes” OR “cognitive processing” OR “cognitive performance” OR “cognitive function” OR “cognitive functions”) AND (“cognitive dysfunction” OR “neurocognitive disorders” OR “cognitive impairment syndrome” OR “early cognitive decline” OR “mild cognitive changes” OR “minor cognitive impairment”) AND (“occupational therapy” OR “occupational therapy interventions” OR “occupational therapist”) AND (“older adults” OR “older people” OR “older subject” OR “aging” OR “ageing” OR “aged”).

Two independent experts were consulted regarding the included articles and the inclusion and exclusion criteria in order to help find more relevant studies. We established two requirements that the experts had to meet: (i) hold a Ph.D. in health sciences or sport sciences; and (ii) have peer-reviewed works published in journals with an impact factor, according to Journal Citation Reports^®^, on physical activity and cognition function in various population groups and/or physical performance. In order to avoid bias in their searches, our search approach was not revealed to the experts. After completing these procedures, on 30 October 2024, we performed a search of the database to find relevant retractions or errata related to the included articles.

### 2.4. Study Selection and Data Collection Process

The studies were exported to Mendeley Reference Manager (version 2.116.1), and the study selection process is illustrated in the PRISMA flowchart. Two authors (EVC and JHM) independently conducted the searches, systematically reviewing titles, abstracts, and full texts, while duplicates were removed. At this stage, no discrepancies were identified. Subsequently, potentially eligible articles were thoroughly re-examined, and exclusions were justified for those that did not meet the predefined selection criteria. Finally, two additional reviewers (CSG and PMM) independently audited the entire selection and data extraction process.

### 2.5. Methodological Quality Assessment

The level of evidence and methodological quality of the studies were evaluated based on the Oxford Centre for Evidence-Based Medicine scale for clinical oncology [23]. Only level 1a studies, defined as RCTs, were included. Studies classified as levels of evidence 1b, 2a, 2b, 3a, 3b, 4, and 5 were excluded. RCTs were downgraded if concerns were raised regarding risk of bias, consistency, accuracy, precision, transparency of results, or publication bias [23].

### 2.6. Data Collection Process

Relevant data were extracted from each study included in the systematic review with meta-analysis and recorded using a data extraction form, following Cochrane recommendations [21], with Microsoft Excel^®^ software (version 16.81). The data extraction was conducted independently by two researchers (EVC and JHM), who subsequently compared the results of their individual analyses. The entire extraction process was jointly overseen by (CSG and PMM). The extracted variables from each study included title, author/year, country of origin, level of evidence, study design, risk of bias, population and sample size, inclusion criteria, study setting, intervention and control groups, outcome measures, and results (Table 2).

### 2.7. Risk of Bias Assessment

The risk of bias in the RCTs included in this review was assessed using the ROB_2_ tool [21]. Two authors (EVC and PVB) independently conducted the analysis, which was subsequently reviewed by two additional authors (CSG and PMM). Any inconsistencies in the initial analyses were addressed through re-examination of the original articles, and disagreements were resolved through consensus.

### 2.8. Measures for Meta-Analysis

The study methodology incorporated a meta-analysis, with full details available on PROSPERO (CRD42023472129). The standardized mean difference (SMD), a statistic that measures the absolute difference between mean values in two groups within an RCT, was calculated for each analysis using Comprehensive Meta-analysis Software (RevMan 5.4). A *p*-value < 0.05 was considered statistically significant [24]. For each trial, a random-effects model, based on the Der Simonian–Laird approach, was employed to calculate and pool the SMD and mean difference (MD) in ADL, cognitive function, and physical function from pre- to post-intervention, comparing experimental and control groups [25]. The underlying assumption of the random-effects model is that true effects, such as intervention type or duration, vary across studies, with data drawn from populations with differing effect sizes. Data were pooled if consistent results were obtained from at least three studies [26]. Heterogeneity among trial results was assessed using the Cochrane Q test [27] and the I^2^ statistic, where I^2^ values of <25%, 25–50%, and >50% represent small, moderate, and substantial inconsistency, respectively [25]. Additionally, Egger’s regression tests were conducted to detect small study effects and potential publication bias [28].

### 2.9. Certainty of Evidence

The certainty of evidence from the included studies was evaluated using the GRADE (Grading of Recommendations, Assessment, Development, and Evaluation) framework [21,29]. Evidence was categorized as high, moderate, low, or very low. All analyses initially started with a high certainty, given the inclusion of RCTs, but were downgraded if concerns arose regarding risk of bias, consistency, accuracy, precision, transparency of results, or publication bias. Two authors (EVC and JHM) independently assessed the studies, with any discrepancies resolved through consensus with the authors (CSG, PMM, and PVB).

## 3. Results

**Figure 1:**
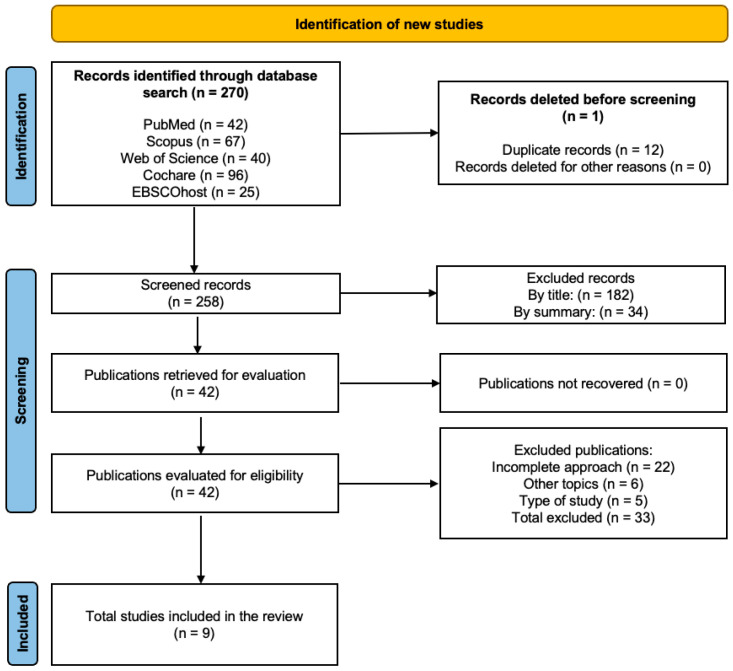
Flowchart of the systematic review.

A total of 270 studies were identified through the database search, with 12 excluded due to duplication. Of the remaining 258 records, 216 were excluded after reviewing titles and abstracts for relevance. Following a full-text review of the 42 references, 33 studies were excluded for not meeting the predetermined inclusion criteria, 22 for including incomplete approaches, six for unrelated topics, and five for not being RCTs. Ultimately, nine studies [30,31,32,33,34,35,36,37,38] were analyzed using the PICOS framework. The search results are presented in a flowchart in accordance with the PRISMA guidelines, as shown in Figure 1 [39].

### 3.1. Methodological Quality

The quality of evidence of the studies included in this systematic review with meta-analysis is rated as high. All nine studies are RCTs [30,31,32,33,34,35,36,37,38], which represent the highest level of evidence (level 1a) according to the Oxford Scale. This study design minimizes the risk of bias, offering a solid basis for reliably assessing the effectiveness of the interventions.

### 3.2. Risk of Bias

**Figure 2:**
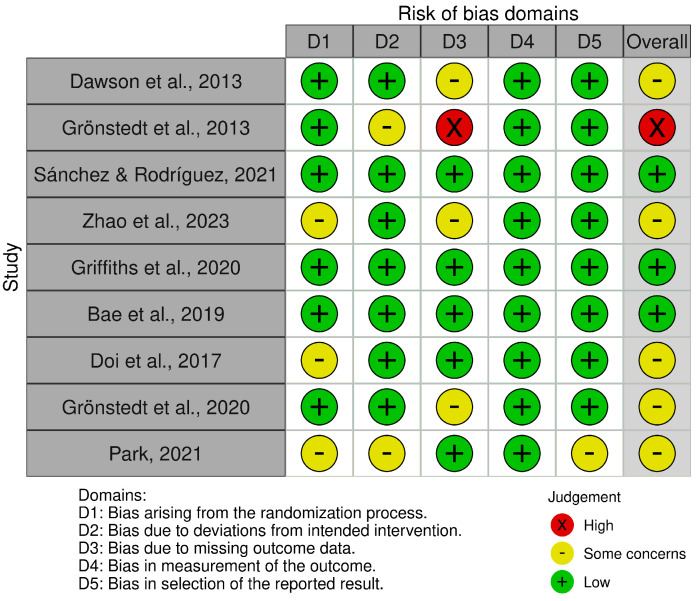
Risk of bias tools: traffic lights chart [30,31,32,33,34,35,36,37,38].

**Figure 3:**
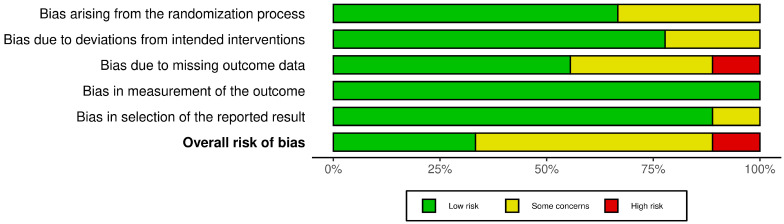
Risk of bias tools: summary chart by domain.

Three studies were assessed as having a low risk of bias [32,34,35], while five studies were found to have some concerns regarding bias [30,33,36,37,38]. One study was classified as having a high risk of bias [31]. Overall, this suggests a moderate risk of bias across the studies, as most exhibited some concerns and one demonstrated a high risk. Figure 2 and Figure 3 provide a summary of the risk of bias assessments.

### 3.3. Characteristics of the Studies

Among the nine studies reviewed, interventions varied, including individualized exercise programs, daily cognitive training, combined aerobic and resistance exercises, and multicomponent training approaches incorporating cognitive elements. For instance, Dawson et al. [30] reported improvements in verbal fluency using self-management education and metacognitive strategies, while Grönstedt et al. [31] observed enhanced balance and physical activity in participants with MCI following an individualized exercise program. Sánchez and Rodríguez [32] documented significant cognitive performance gains from daily cognitive training. Zhao et al. [33] found that combined aerobic and resistance exercises improved cognitive performance in sedentary older people. Similarly, Griffiths et al. [34] demonstrated improvements in attention and executive function after implementing a combined physical and cognitive training program. Bae et al. [35] underscored the benefits of a multicomponent intervention in promoting both physical activity and cognitive outcomes. Doi et al. [36] showed that cognitive leisure activities, such as dance and music, led to higher cognitive scores compared to health education. Grönstedt et al. [37] highlighted the positive effects of sit-to-stand exercises and nutritional supplementation on nutritional status and physical performance. Finally, Park [38] reported that dual-task cognitive–physical training improved executive function, as measured by Trail Making Test B in older people with MCI. Detailed descriptions of each study and their outcome measures are provided in Table 2.

30sCST: 30-Second Chair Stand Test; 6-MWT: 6-Minute Walk Test; ADL: Activities of Daily Living; BD: Block Design; CA: Canada; CG: control group; COPM: Canadian Occupational Performance Measure; CST: Chair Stand Test; CVF: Category Verbal Fluency; D-KEFS: Delis–Kaplan Executive Function System; DSB: Digit Span Backward; DSF: Digit Span Forward; DSS: Digit Span Sequencing; ECB: Everyday Cognition Battery; EG: experimental group; EQ5D-5L: European Quality of Life 5 Dimensions; ERFC: Rapid Assessment of Cognitive Functions; FIM: Functional Independence Measure; KR: Korea; IADL: Instrumental Activities of Daily Living; LVF: Letter Verbal Fluency; MCI: Mild Cognitive Impairment; MMSE: Mini-Mental State Examination; MVPA: Moderate To Vigorous Physical Activity; n: Sample Size (number of participants); NCGG-FAT: National Center For Geriatrics And Gerontology Functional Assessment Tool; NR: Not Reported; RCT: randomized controlled trial; SP: Spain; T2DM: Diabetes Mellitus; TH: Thailand; TMT-A: Trail Making Test A; TMT-B: Trail Making Test B; TMT: Trail Making Test; TW: Taiwan; USA: United States of America; WLL Delayed: Wordlist Learning Delayed Recall; WLL Imm: Wordlist Learning Immediate Recall.

**Table 2:** Characteristic of the included studies.

Study	Country or Multicenter	Study Design	Sample	Groups (n)	Mean Age (Years)	Type of Intervention and Control Group	Training Volume			Training Intensity	Cognitive Function (Assessment)	Physical Function (Assessments)	**ADL** **(Assessments)**	**Main Outcomes**
							Weeks	Frequency (Sessions /Week)	Session Duration (Minutes)					
Dawson et al. [30]	CA	RCT	Healthy older adults with cognitive complaints	EG: 10 CG: 9	EG: 74.10 (8.77), 90% female. CG: 73.67 (5.43), 78% female	EG: education about self-management and occupation-based meta-cognitive strategy training CG: education brain health and cognitively stimulating exercises	8	2	60	Moderate	General Self-Efficacy Scale, D-KEFS Tower Test, verbal fluency	Stanford Chronic Disease Questionnaire	COPM	Both groups: ↔ ADL (COPM) (*p* = 0.54) EG: ↑ Word fluency (*p* = 0.01) ↑ Untrained everyday life problems (*p* = 0.03) CG: ↓ Communication with physicians (*p* = 0.02) ↓ Physical activity (*p* = 0.02)
Grönstedt et al. [31]	Multicenter	RCT	Subjects diagnosed with MCI	EG: 170 CG: 152	EG: 85, 71% female CG: 87.74, 76% female	EG: individual physical training and group activities such as outdoor walks and personal care, clothing and nutrition GC: standard care (without a specific focus on physical rehabilitation)	10	3	30	Moderate	MMSE	Berg Balance Scale, timed CST, Short Falls Efficacy Scale	FIM	Both groups: ↔ ADL (FIM) (*p* = 0.293) EG: ↑ Balance (*p* = 0.001) ↑ Physical activity (*p* = 0.038) ↑ Transfers (*p* = 0.024) CG: ↓ ADL (*p* = 0.012) ↓ Balance (*p* = 0.004) ↓ Deterioration in transfers (*p* = 0.023)
Sánchez & Rodríguez [32]	SP	RCT	Older adults with MCI	EG: 137 CG: 130	EG: 73.89, 83.90% female CG: 72.99, 83.10% female	EG: Everyday Cognition Training Program CG: conventional cognitive training program	10	2	50	Moderate	ERFC	NR	ECB	EG: ↑ Cognitive performance (ERFC) between 1-PRE and 8-POST (*p* < 0.001) ↑ ECB between 1-PRE and 8-POST (*p* < 0.001) CG: ↓ Cognitive performance (ERFC) between 1-PRE and 8-POST (*p* < 0.001) ↓ ECB between 1-PRE and 8-POST (*p* < 0.001)
Zhao et al. [33]	USA	RCT	Older sedentary adults with T2DM and cognitive impairment	EG: 32 CG: 40	EG: 66.1, 50% female GC: 65.9, 50% female	EG: combined aerobic and resistance exercise program CG: sedentary healthy older adults with no specific intervention	10	3	30	Moderate	Mini-Cog, TMT	NR	NR	Cognitive Performance: ↔ Mini-Cog (*p* = 0.0005) ↔ TMT-A (*p* = 0.006) ↔ TMT-B (*p* < 0.001) EG: ↑ Mini-Cog scores (*p* = 0.005) ↑ TMT-A (*p* = 0.006) ↑ TMT-B (*p* < 0.001) CG: ↓ Mini-Cog performance (*p* = 0.005) ↓ TMT-A (*p* = 0.006) ↓ Deterioration in TMT-B (*p* < 0.001) Physical Fitness Outcomes: Both Groups: ↔ 6-MWT (*p* = 0.293) EG: ↑ 6-MWT distance (*p* < 0.01)
Griffiths et al. [34]	TH	RCT	Older adult and people with MCI	EG: 35 CG: 35	EG: 65.14, 74% female CG: 67.23, 72% female	EG: combined physical movement activity and multifaceted cognitive training CG: waitlist control (standard care)	12	2	NR	Moderate	TMT, DSF, DSB, DSS	10 m walking, grip strength, timed CST	NR	EG: ↑ Attention (TMT-A) (*p* = 0.023) ↑ Executive function (BD) (*p* = 0.029) ↑ LVF (*p* = 0.001) ↑ CVF (*p* = 0.004) ↑ WLL imm (*p* = 0.023) ↑ WLL delayed (*p* = 0.036) CG: ↔ Stable performance in attention (TMT-A) (*p* = 0.293) ↔ No significant changes in executive function (BD) (*p* = 0.036) ↔ Deterioration in verbal fluency (LVF) (*p* = 0.036) ↔ Deterioration in WLL imm (*p* = 0.012)
Bae et al. [35]	Multicenter	RCT	Adults diagnosed with MCI	EG: 41 CG: 42	EG: 76.4, 61% female CG: 75.5, 43.9% female	EG: multicomponent intervention (community activity program) CG: health education classes	24	2	90	Moderate to vigorous	NCGG-FAT (memory, attention, executive function, processing speed), MMSE, Word Recall Test, TMT	Grip strength, 10 m walking speed, timed CST, MVPA, step count	NR	EG: ↑ TMT-A (*p* = 0.001) ↑ Working memory scores (*p* = 0.010) ↑ MVPA (*p* = 0.048) ↑ Step count (*p* = 0.059) CG: ↔ Walking speed (*p* = 0.099) ↔ Grip strength (*p* = 0.136) ↔ MMSE (*p* = 0.434) EG: **↑** TMT-A (*p* = 0.001) **↑** Working memory scores (*p* = 0.010) **↑** MVPA (*p* = 0.048) **↑** Step count (*p* = 0.059) CG: ↔ Walking speed (*p* = 0.099) **↔** Grip strength (*p* = 0.136) **↔** MMSE score (*p* = 0.434)
Doi et al. [36]	JP	RCT	Subjects diagnosed with MCI	EG: 109 CG: 67	EG: 75.7, 50.7% female 76.0% 58.2% female	EG: cognitive leisure activities (dance or music) CG: health education	40	1	60	Low	TMT-A, TMT-B, MMSE	NR	NR	EG: ↑ MMSE scores compared to CG (dance: *p* = 0.026, music: *p* = 0.008) ↔ TMT-A and TMT-B scores compared to the CG
Grönstedt et al. [37]	Multicenter	RCT	Older adults with MCI	EG: 35 CG: 35	EG: 85.9, 62% female CG: 85.9, 58% female	EG: sit-to-Stand exercises in conjunction with the ADLs combined with protein-rich oral supplementation CG: standard care (without a specific focus on physical rehabilitation)	12	7	Variable (integrated into daily activities)		MMSE	10 m walking speed, grip strength, timed CST	FIM	Both groups ↔ 30sCST (*p* = 0.325) ↔ ADL (FIM) (*p* = 0.55) ↔ Quality of Life (EQ5D-5L) (*p* = 0.59) EG: ↑ Nutritional status (*p* = 0.007) ↑ Fat-free mass (*p* = 0.007) CG: ↓ Balance (Berg Balance Scale) (*p* = 0.10) ↓ Walking speed (*p* = 0.10)
Park [38]	KR	RCT	Older adults with MCI	EG: 18, CG: 18	EG: 74.00, 50% female; CG: 74.00, 50%female	EG: cognitive–physical dual-task training; CG: single cognitive training focused on executive function	8	2	40	Moderate	TMT-B	NR	K-IADL	Both groups: ↔ K-IADL (*p* > 0.05) EG: ↑ TMT-B performance (*p* < 0.001) ↓ PFC activity during TMT-B (*p* < 0.001) CG: ↑ TMT-B performance (*p* < 0.001) ↓ PFC activity during TMT-B (*p* < 0.001)

↔: No changes; ↑: Increased or higher; ↓: Decreased or lower. However, not explanation should be added; 30sCST: 30-Second Chair Stand Test; 6-MWT: 6-Minute Walk Test; ADL: Activities of Daily Living; BD: Block Design; CA: Canada; CG: control group; COPM: Canadian Occupational Performance Measure; CST: Chair Stand Test; CVF: Category Verbal Fluency; D-KEFS: Delis–Kaplan Executive Function System; DSB: Digit Span Backward; DSF: Digit Span Forward; DSS: Digit Span Sequencing; ECB: Everyday Cognition Battery; EG: experimental group; EQ5D-5L: European Quality of Life 5 Dimensions; ERFC: Rapid Assessment of Cognitive Functions; FIM: Functional Independence Measure; KR: Korea; IADL: Instrumental Activities of Daily Living; LVF: Letter Verbal Fluency; MCI: Mild Cognitive Impairment; MMSE: Mini-Mental State Examination; MVPA: Moderate To Vigorous Physical Activity; n: Sample Size (number of participants); NCGG-FAT: National Center For Geriatrics And Gerontology Functional Assessment Tool; NR: Not Reported; RCT: randomized controlled trial; SP: Spain; T2DM: Diabetes Mellitus; TH: Thailand; TMT-A: Trail Making Test A; TMT-B: Trail Making Test B; TMT: Trail Making Test; TW: Taiwan; USA: United States of America; WLL Delayed: Wordlist Learning Delayed Recall; WLL Imm: Wordlist Learning Immediate Recall.

### 3.4. Sample Characteristics

The total population included in this systematic review with meta-analysis comprised 752 participants, with 64.5% being female, and a mean age of 75.3 years. Sample sizes ranged from 18 [38] to 170 [31] participants, reflecting the diversity of interventions. These interventions, which included cognitive training, individualized exercise programs, and multicomponent approaches, were assessed for their effects on ADL, cognitive function, and physical function in older people with MCI.

### 3.5. Dosages and Interventions Performed

The studies included a range of cognitive and physical interventions aimed at enhancing cognitive function and ADLs among MCI. Cognitive training programs, such as daily cognitive exercises and leisure activities, primarily targeted cognitive performance [32,36]. Individualized exercise programs and multicomponent interventions, which combined physical activity with cognitive training, were shown to significantly improve both cognitive function and ADL performance [31,34]. These studies implemented interventions with varying durations and frequencies, from eight weeks with two 60-minute sessions per week to protocols lasting 26 weeks with multiple sessions combining from 10 to 90 minutes each [30,31,32,33,34,35,36,37,38]. Most programs were conducted at moderate intensity.

### 3.6. Activities of Daily Living

No significant changes were observed in favor of the EG compared to the CG. A meta-analysis on ADL outcomes was not feasible due to the limited number of relevant studies and the use of varied assessment methods. Only three studies assessed ADL interventions with validated tools. Grönstedt et al. [37] reported significant ADL improvements in older adults using a sit-to-stand exercise program with nutritional supplements (*p* < 0.05). Park [38] found similar improvements in older adults with MCI using a multicomponent intervention combining physical and cognitive elements (*p* < 0.05). However, Liu et al. [40] found no significant ADL changes (*p* = 0.423), underscoring the need for standardized assessment protocols to clarify intervention impacts.

### 3.7. Cognitive Function

**Figure 4:**
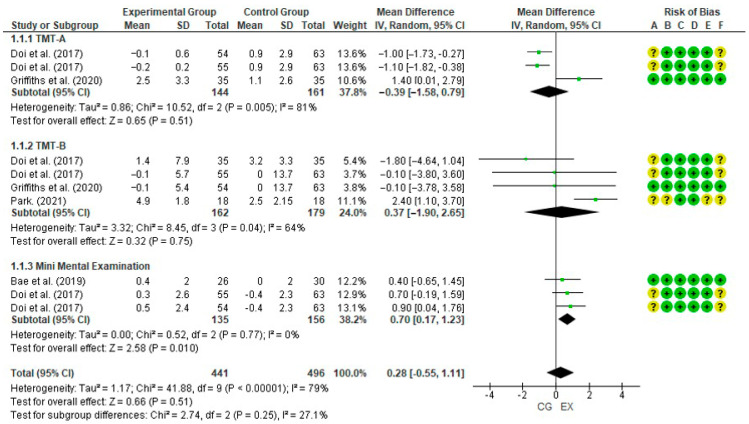
Experimental group has an effect compared to control group on the following outcomes: Trail Making Test A (TMT-A), Trail Making Test B (TMT-B), and Mini-Mental Examination. Squares indicate the study-specific effect estimate. Bars indicate the width of the corresponding 95% confidence interval. The diamond/rhombus is centered on the summary effect estimate, and the width indicates the corresponding 95% confidence interval. EX: experimental group; CG: control group; +: low; ?: some concerns [34,35,36].

Four studies employed the MMSE to measure cognitive improvements, with one meta-analysis showing a significant improvement in favor of the EG (SMD = 0.70; 95% CI = 0.17 to 1.23; I^2^ = 0%; *p* = 0.010). In contrast, the meta-analyses of TMT-A (SMD = −0.39; 95% CI = −1.58 to 0.79; I^2^ = 81%; *p* = 0.51) and TMT-B (SMD = 0.37; 95% CI = −1.90 to 2.65; I^2^ = 64%; *p* = 0.37) did not reveal significant improvements in favor of the EG, highlighting the variability between studies in terms of efficacy. The results of the meta-analysis are presented in Figure 4.

The individual results of the studies indicate notable cognitive improvements across various interventions. Dawson et al. [30] reported significant gains in verbal fluency (*p* < 0.05) following occupation-based metacognitive strategy training. Park [38] observed enhanced executive function after a dual-task training program (*p* < 0.05). Similarly, Sánchez and Rodríguez [32] documented cognitive gains with daily cognitive training (*p* < 0.01), and Zhao et al. [33] found significant improvements (*p* < 0.01) from combined aerobic and resistance exercise. Additionally, Doi et al. [36] reported cognitive benefits (*p* < 0.001) from leisure-time cognitive activities.

### 3.8. Physical Function

A meta-analysis was planned, but it could not be performed due to heterogeneous assessments. The reviewed studies highlighted positive effects of structured physical interventions on physical function in older people with MCI. No changes in favor of experimental group (EG) with respect to control group (CG) could be observed. However, Grönstedt et al. [37] observed significant improvements (*p* < 0.01) in balance and physical activity in participants who performed individualized exercises. Zhao et al. [33] demonstrated that a combined aerobic and resistance exercise program significantly improved physical performance in (*p* < 0.01) older people with MCI. Park [38] reported significant improvements in ADL performance (*p* < 0.05) after a multicomponent intervention combining physical and cognitive training.

### 3.9. Certainty of Evidence

The certainty of evidence was insufficient to support definitive recommendations for interventions aimed at improving ADL, cognitive function, and physical function in older people with MCI. While some studies demonstrated promising outcomes in these domains, the overall findings underscore the need for further research to establish clearer conclusions and guide evidence-based interventions in this population (Table 3).

### 3.10. Adverse Effects and Adherence

The studies included in this systematic review with meta-analysis reported adequate adherence from the participants but did not report any adverse effects. This suggests that, overall, the interventions were well tolerated and feasible for older people with MCI, supporting their potential for broader implementation in similar populations.

## 4. Discussion

The evidence highlights the effectiveness of integrating cognitive and physical interventions to improving both cognitive function and physical capabilities in older adults, particularly those with MCI. Studies show that multicomponent or dual-task interventions significantly enhance global cognition, executive function, memory, and ADL, outperforming single-domain interventions. Improvements in ADL and physical functions, such as balance, strength, and mobility, are linked to neuroplasticity, which is stimulated by the combination of cognitive and physical tasks. While traditional physical activity alone shows limited impact, the synergy of cognitive stimulation with physical exercise demonstrates superior outcomes, supporting the importance of holistic, multidomain strategies in promoting functional independence and slowing cognitive decline in older populations.

### 4.1. Activities of Daily Living

A meta-analysis of studies on ADL outcomes was not feasible due to limited studies and varied assessment methods, with no significant differences found between the EG and CG. Bae et al. [35] and Park [38] reported significant improvements in ADL among participants following multicomponent or dual-task interventions that integrated cognitive and physical activities. Bae et al. [35] implemented a 6-week program of cognitive stimulation paired with physical activity, while Park [38] used a multicomponent intervention combining physical and cognitive training. This is consistent with the existing literature that emphasizes how multitasking interventions not only enhance physical fitness but also strengthen executive function, further supporting the importance of integrative approaches in maintaining ADL capabilities [41]. Dual-task interventions not only improve physical fitness but also enhance the ability to perform ADLs, reinforcing the significance of integrative strategies in promoting functional independence among older people [42]. Combined interventions of physical activity and cognitive stimulation have been shown to be effective in improving functioning in older people with MCI, improving both cognitive and physical functions and their independence in ADL [43]. The intervention dosage should be standardized in future studies to allow for more precise comparisons. It is known that increased intensity, both in session duration and the number of weeks, could lead to better functional outcomes [44].

Repetition of ADLs promotes neuroplasticity, facilitating the formation of new neural connections. This process is key in functional rehabilitation, allowing healthy brain areas to take over functions from damaged regions, which improves the independence of patients [45].

### 4.2. Cognitive Function

The meta-analysis revealed significant improvements in the MMSE in favor of the EG concerning CG. No significant improvements were found for TMT-A (*p* = 0.51) or TMT-B (*p* = 0.37). Huang et al. [46] report in their meta-analysis that while endurance exercise is the most promising intervention for slowing cognitive decline in older people with MCI, multicomponent exercise may offer superior benefits for preserving global cognition (*p* < 0.04), executive function (*p* < 0.05), and memory (*p* < 0.07) in older people with MCI. Meng et al. [47] report in another meta-analysis that combined intervention showed a significant improvement in global cognition (*p* = 0.003), memory (*p* = 0.001), and executive function/attention (*p* = 0.004). This cautions that the combination may be superior to individual cognitive or physical interventions. In their meta-analysis on physical activity, Biazus-Sehn et al. [48] demonstrated cognitive improvements in older people with MCI, especially with mind–body and moderate-intensity exercises. The results showed significant improvements in global cognitive function (*p* = 0.0001), executive function (*p* = 0.026), and delayed recall (*p* = 0.047). No significant effects on verbal fluency (*p* = 0.069) and attention (*p* = 0.073) were also observed. Salzman et al. [49] report in another meta-analysis that multidomain interventions demonstrated significant cognitive improvements across multiple domains in older people with MCI, specifically, significant effects on global cognition (*p* < 0.001), memory (*p* < 0.001), and executive function (*p* = 0.01). These findings suggest that combining cognitive training with physical activity may be beneficial for cognitive function in this population.

### 4.3. Physical Function

Meta-analysis was not possible due to the different assessment guidelines used. Outcomes related to physical function following an intervention in older people with MCI varied between studies. Griffiths et al. [34] and Grönstedt et al. [37] both reported that combined aerobic, resistance, and physical exercises significantly improved (*p* < 0.05) balance, muscle strength, and functional mobility. In contrast, studies that utilized only traditional physical activity without cognitive components, such as Henskens et al. [50], did not demonstrate significant improvements in physical function (*p* = 0.114). Henskens et al. [50] employed a standard 12-week resistance training program that lacked cognitive elements. These findings suggest that interventions combining cognitive and physical activities may be more effective for improving physical function in older people, potentially supporting better health outcomes and extended periods of independence.

The evidence points to a clear advantage in developing multifaceted intervention strategies that target both cognitive and physical domains to optimize functional abilities in aging populations [51]. Castellote-Caballero et al. [52] report that integrated interventions, combining physical activity and cognitive training, are crucial to address both physical and cognitive health in older people, suggesting that this dual approach is a promising therapeutic strategy to mitigate age-related decline and improve overall quality of life. Integrating physical activity into the treatment of MCI improves cognitive performance and brain health by increasing brain-derived neurotrophic factor, which promotes neurogenesis and enhances cognitive functions such as memory and learning [53]. This increase in neuroplasticity helps maintain and potentially restore cognitive function, offering a promising strategy to reduce cognitive decline in MCI.

### 4.4. Strengths and Limitations

This systematic review with meta-analysis has some limitations: (i) the lack of statistically significant improvements in physical function and ADLs in the studies limits the ability to generalize the effectiveness of the interventions; (ii) variability in intervention designs, including differences in dosage, duration, and types of activities, complicates the generalizability of the findings; and (iii) high heterogeneity in cognitive function assessment results, particularly in TMT-A (I^2^ = 81%), highlights the need for further research to clarify the factors influencing these outcomes. The strengths of the systematic review with meta-analysis include the following: (i) the inclusion of diverse interventions highlights the benefits of combining cognitive and physical activities, as evidenced by significant improvements in MMSE scores; (ii) most interventions are aligned with the current literature, which emphasizes the importance of multitasking to improve functional skills in older people; and (iii) several articles present standardized protocols detailing intervention dosage, including duration and intensity, which facilitates the replication of successful strategies and optimizes evidence-based programs.

### 4.5. Practical Applications

This systematic review and meta-analysis evaluate the effects of combined cognitive stimulation and physical activity interventions on older adults with MCI. The results, however, show inconsistencies in improvements related to ADLs and cognitive function, with diverse outcomes across the included studies [34,36,40]. The discrepancies highlight the need for standardized intervention protocols that include clear criteria regarding the duration, intensity, and types of interventions to improve consistency and transparency in implementation. Moreover, the incorporation of innovative delivery methods, particularly the use of technology in cognitive and physical activity therapies, may improve engagement and overall effectiveness, as demonstrated by Zhao et al. [33]. Long-term follow-up studies, ideally spanning 12 to 24 weeks, are essential to ascertain the sustainability of the observed improvements in Activities of Daily Living (ADLs) and cognitive function. This highlights the significance of culturally appropriate, multitask interventions, particularly in light of the increasing prevalence of MCI among the aging population.

## 5. Conclusions

The combined physical activity and cognitive stimulation interventions led to significant improvements in cognitive function, as assessed by the MMSE. However, these interventions did not yield significant improvements in ADL or physical function among older adults with MCI. Individual studies suggest that programs that integrate both cognitive stimulation and physical activity components may provide some benefits. To establish more consistent findings and optimize the design of effective interventions aimed at improving cognitive function and preserving independence in older people, further research is needed. This research should focus on specifying the dose, duration, and components of these interventions to better understand their impact and replicate in the future.

## Figures and Tables

**Table 1:** Criteria of selection used in the systematic review.

Category	Inclusion	Exclusion
Population	Studies were included if they involved populations with a mean age of 60 years or older, with a diagnosis of MCI	Studies with populations whose main pathology was unrelated to MCI (i.e., chronic diseases, physical deterioration, or social problems).
Intervention	Studies involving OT interventions or programs combined with cognitive stimulation and physical activity	Studies whose focus is on interventions unrelated to OT interventions or cognitive stimulation and physical activity
Comparator	Interventions with active or inactive control groups	Studies lacking control groups or having only inactive control groups
Outcome	At least one assessment of ADL, cognitive function, or physical function.	Studies without baseline data and/or follow-ups
Study design	Randomized controlled trials, with pre- and post-assessment	Non-randomized, cross-sectional, retrospective, and prospective controlled studies
Level of evidence	1a	1b, 2a, 2b, 3a, 3b, 4, and 5

ADL: Activities of Daily Living; MCI: Mild Cognitive Impairment; OT: Occupational Therapist.

**Table 3:** Methodological quality assessment using GRADEpro tool.

Certainty of Evidence							Nº of Patients		Effect		Certainty	Importance
Nº of Studies	Study Design	Risk Assessment	Inconsistency	Indirect Evidence	Vagueness	Other Considerations	[Conventional Therapy Plus Virtual Reality]	[Conventional Therapy]	Relative (95% CI)	Absolute (95% CI)		
**An occupation-based strategy training approach to managing age-related executive changes: a pilot randomized controlled trial**												
1	RCT	Serious	It is not serious	It is not serious	It is not serious	None	10/19 (52.6%)	9/19 (47.4%)	Not estimable		+++ Moderate	IMPORTANT
**Effects of Individually Tailored Physical and Daily Activities in Nursing Home Residents on Activities of Daily Living, Physical Performance and Physical Activity Level: A Randomized Controlled Trial**												
1	RCT	Very serious	It is not serious	It is not serious	It is not serious	None	170/322 (52.8%)	152/322 (47.5%)	Not estimable		++ Low	IMPORTANT
**The Effectiveness of a Training Program in Everyday Cognition in Healthy Older Adults: A Randomized Controlled Trial**												
1	RCT	It is not serious	It is not serious	It is not serious	It is not serious	None	132/267 (51.3%)	130/267 (48.7%)	Not estimable		++++ High	IMPORTANT
**Operational Modal Analysis of Near-Infrared Spectroscopy Measure of 2-Month Exercise Intervention Effects in Sedentary Older Adults with and Cognitive Impairment**												
1	RCT	Serious	It is not serious	It is not serious	It is not serious	None	32/72 (44.4%)	40/72 (55.6%)	Not estimable		+++ Moderate	IMPORTANT
**Effects of Combined Physical Movement Activity and Multifaceted Cognitive Training in Older People with Mild Neurocognitive Disorder in a Rural Community: A Randomized Control Trial**												
1	RCT	It is not serious	It is not serious	It is not serious	It is not serious	None	35/70 (50%)	35/70 (50%)	Not estimable		++++ High	IMPORTANT
**The Effect of a Multicomponent Intervention to Promote Community Activity on Cognitive Function in Older Adults with MCI: A randomized controlled trial**												
1	RCT	It is not serious	It is not serious	It is not serious	It is not serious	None	41/83 (49.4%)	42/83 (50.6.%)	Not estimable		++++ High	IMPORTANT
**Effects of Cognitive Leisure Activity on Cognition in Mild Cognitive Impairment: Results of a Randomized Controlled Trial**												
1	RCT	Serious	It is not serious	It is not serious	It is not serious	None	109/176 (61.9%)	67/176 (38.1%)	Not estimable		+++ Moderate	IMPORTANT
**Effects of 6-Month Combined Physical Exercise and Cognitive Training on Neuropsychological and Neurophysiological Function in Older Adults with Subjective Cognitive Decline: A Randomized Controlled Trial**												
1	RCT	Serious	It is not serious	It is not serious	It is not serious	None	35/70 (50.0%)	35/70 (50.0%)	Not estimable		+++ Moderate	IMPORTANT
**Effects of Cognitive–Physical Dual-Task Training on Executive Function and Activity in the Prefrontal Cortex of Older Adults with Mild Cognitive Impairment**												
1	RCT	Serious	It is not serious	It is not serious	It is not serious	None	18/36 (50.0%)	18/36 (50.0%)	Not estimable		+++ Moderate	IMPORTANT

ADL: Activities of Daily Living; CI: confidence interval; MCI: Mild Cognitive Impairment; RCT: randomized controlled trial.

## Data Availability

Not applicable.
